# Supplementary material for: A Qualitative Study Exploring Professional Perspectives of a Challenging Rehabilitation Environment for Geriatric Rehabilitation
Source: J Clin Med. 2023 Feb 3;12(3):1231. doi: 10.3390/jcm12031231 (PMC9918066; doi:10.3390/jcm12031231)
Supplement: Supplementary file 1 [file jcm-12-01231-s001.zip › jcm-2106121-supplementary.pdf]

Supplemental Table S1

COREQ (Consolidated criteria for Reporting Qualitative research) Checklist

**Topic**	**Item No.**	**Guide Questions/Description**	**Reported online No.**
**Domain 1: Research team and reflexivity**			
Personal characteristics			
Interviewer/facilitator	1	Which author/s conducted the interview or focus group?	124
Credentials	2	What were the researcher's credentials? E.g. PhD, MD	124-128
Occupation	3	What was their occupation at the time of the study?	124-128
Gender	4	Was the researcher male or female?	124-128
Experience and training	5	What experience or training did the researcher have?	124-128
Relationship with participants			
Relationship established	6	Was a relationship established prior to study commencement?	138-139<br>150-152
Participant knowledge of the interviewer	7	What did the participants know about the researcher? E.g., personal goals, reasons for doing the research	138-139
Interviewer characteristics	8	What characteristics were reported about the interviewer/facilitator? E.g., bias, assumptions, reasons and interests in the research topic	124-128
**Domain 2: Study design**			
Theoretical framework			
Methodological orientation and Theory	9	What methodological orientation was stated to underpin the study? E.g., grounded theory, discourse analysis, ethnography, phenomenology, content analysis	79-84
Participant selection			
Sampling	10	How were participants selected? E.g., purposive, convenience, consecutive, snowball	90-121
Method of approach	11	How were participants approached? E.g., face-to-face, telephone, mail, email	90-121
Sample size	12	How many participants were in the study?	174-176
Non-participation	13	How many people refused to participate or dropped out? Reasons?	176-180
Setting			
Setting of data collection	14	Where was the data collected? E.g., home, clinic, workplace	133-137<br>149-150
Presence of non-participants	15	Was anyone else present besides the participants and researchers?	137
Description of sample	16	What are the important characteristics of the sample? E.g., demographic data, date	Table 1
Data collection			
Interview guide	17	Were questions, prompts, guides provided by the authors? Was it pilot tested?	129-132<br>Appendix 1
Repeat interviews	18	Were repeat interviews carried out? If yes, how many?	113-115<br>174-176
Audio/visual recording	19	Did the research use audio or visual recording to collect the data?	144-145<br>154-156

Field notes	20	Were field notes made during and/or after the interview or focus group?	124
Duration	21	What was the duration of the interviews or focus group?	144<br>149
Data saturation	22	Was data saturation discussed?	113-115
Transcripts returned	23	Were transcripts returned to participants for comment and/or correction?	145-147
**Domain 3: analysis and findings**			
Data analysis			
Number of data coders	24	How many data coders coded the data?	158-164
Description of the coding tree	25	Did authors provide a description of the coding tree?	158-171
Derivation of themes	26	Were themes identified in advance or derived from the data?	158-171
Software	27	What software, if applicable, was used to manage the data?	159
Participant checking	28	Did participants provide feedback on the findings?	145-147
Reporting			
Quotations presented	29	Were participant quotations presented to illustrate the themes/findings? Was each quotation identified? E.g., participant number	199-338
Data and findings consistent	30	Was there consistency between the data presented and the findings?	199-338 Table 2 and 3
Clarity of major themes	31	Were major themes clearly presented in the findings?	199-338 Table 2 and 3
Clarity of minor themes	32	Is there a description of diverse cases or discussion of minor themes?	199-338 Table 2 and 3
